# Development of an RNA virus vector for non-transgenic genome editing in tobacco and generation of *berberine bridge enzyme-like* mutants with reduced nicotine content

**DOI:** 10.1007/s42994-024-00188-y

**Published:** 2024-11-22

**Authors:** Haiying Xiang, Binhuan Chen, Shuo Wang, Wanli Zeng, Jiarui Jiang, Weisong Kong, Haitao Huang, Qili Mi, Shuang Ni, Qian Gao, Zhenghe Li

**Affiliations:** 1 Yunnan Academy of Tobacco Science, Kunming, 650106 China; 2 State Key Laboratory of Rice Biology, Institute of Biotechnology, Zhejiang University, Hangzhou, 310058 China; 3 Ministry of Agriculture Key Laboratory of Molecular Biology of Crop Pathogens and Insect Pests, Zhejiang University, Hangzhou, 310058 China; 4 Key Laboratory of Biology of Crop Pathogens and Insects of Zhejiang Province, Zhejiang University, Hangzhou, 310058 China

**Keywords:** Eggplant mottled dwarf virus, Rhabdovirus, Delivery, CRISPR/Cas, Genome editing, Berberine bridge enzyme-like, Tobacco, Nicotine, Alkaloid

## Abstract

Tobacco (*Nicotiana tabacum*) plants synthesize the psychoactive pyridine alkaloid nicotine, which has sparked growing interest in reducing nicotine levels through genome editing aiming at inactivating key biosynthetic genes. Although stable transformation-mediated genome editing is effective in tobacco, its polyploid nature complicates the complete knockout of genes and the segregation of transgenes from edited plants. In this study, we developed a non-transgenic genome editing method in tobacco by delivering the CRISPR/Cas machinery via an engineered negative-strand RNA rhabdovirus vector, followed by the regeneration of mutant plants through tissue culture. Using this method, we targeted six *berberine bridge enzyme-like protein* (*BBL*) family genes for mutagenesis, which are implicated in the last steps of pyridine alkaloid biosynthesis, in the commercial tobacco cultivar Hongda. We generated a panel of 16 mutant lines that were homozygous for mutations in various combinations of *BBL* genes. Alkaloid profiling revealed that lines homozygous for *BBLa* and *BBLb* mutations exhibited drastically reduced nicotine levels, while other *BBL* members played a minor role in nicotine synthesis. The decline of nicotine content in these lines was accompanied by reductions in anatabine and cotinine levels but increases in nornicotine and its derivative myosmine. Preliminary agronomic evaluation identified two low-nicotine lines with growth phenotypes comparable to those of wild-type plants under greenhouse and field conditions. Our work provides potentially valuable genetic materials for breeding low-nicotine tobacco and enhances our understanding of alkaloid biosynthesis.

**Supplementary Information:**

The online version contains supplementary material available at 10.1007/s42994-024-00188-y.

## Introduction

The advent of genome editing technologies, particularly those based on CRISPR/Cas systems, offers the potential for rapid and precise genome modifications that are virtually indistinguishable from natural genetic variations, thus holding great promise for accelerated crop improvement (Li et al. 2024; Steinwand and Ronald 2020). As a genome-modifying tool, CRISPR/Cas reagents need only to be transiently present in the plant cells to induce the desired genetic modifications. However, they are typically introduced into plant cells through stable transgene expression, achieved either via *Agrobacterium tumefaciens*-mediated transformation or biolistic particle bombardment (Gao 2021; Ghogare et al. 2021). Transgenesis-based delivery methods, while effective in certain plant species, often result in disruption of essential genes or chromosomal rearrangements (Jupe et al. 2019; Liu et al. 2019a; Thomson et al. 2024; Yue et al. 2022). Although it is possible to segregate the transgene in successive generations through sexual reproduction, this process can be labor-intensive and time-consuming. Therefore, plant genome editing without stable transformation is particularly desired, especially when the edited plants are intended for field trait evaluation, or when targeting multiple gene copies, where transgene segregation may be more challenging due to genetic linkage (Bhattacharjee et al. 2023; Gong et al. 2021).

In recent years, viral vectors have emerged as a promising alternative for delivering CRISPR/Cas components into plants (Shen et al. 2024; Uranga and Daròs 2023). Positive-strand RNA viruses, such as tobacco rattle virus and barley stripe mosaic virus, have been successfully adapted to deliver single guide RNA (sgRNA) to Cas9-expressing plants, enabling heritable gene edits without the need for tissue culture, thus simplifying the editing process (Ellison et al. 2020; Li et al. 2021; Uranga et al. 2023). In addition, we have previously leveraged the large cargo capacities of plant negative-strand RNA viruses, including the rhabdovirus sonchus yellow net virus (SYNV) and the tospovirus tomato spotted wilt virus (TSWV), for delivering the entire CRISPR/Cas machinery to bypass the need for stable transformation in plant genome editing (Liu et al. 2023; Ma et al. 2020; Zhao et al. 2024). Despite the potential of viral delivery approaches, their application in crop species remains limited. To broaden their utility in agriculturally important crops, further development of viral vectors with diverse genetic and biological properties is necessary.

The allotetraploid common tobacco (*Nicotiana tabacum* L.) is widely cultivated as a non-food crop of significant economic importance globally. The pervasive use of tobacco, largely driven by nicotine's highly addictive properties, presents a leading cause of preventable illness and death worldwide. Addressing this public health challenge has spurred efforts to develop tobacco strains with substantially reduced or non-addictive nicotine levels (Guo et al. 2021; Shoji et al. 2024). Nicotine, along with related pyridine alkaloids such as anabasine, anatabine, and nornicotine, are specialized secondary metabolites predominantly found in *Nicotiana* species (Kaminski et al. 2020). Structurally, nicotine is composed of a pyridine ring and a pyrrolidine ring, both originating from amino acid precursors, which then condense in the later steps of the biosynthesis of nicotine (Dewey and Xie 2013; Shoji 2020). While enzymes involved in the early biosynthetic steps that intersect with the polyamine and NAD pathways are well understood, the exact mechanism of ring condensation in the later steps remains unclear. Two oxidoreductases, A622—an NADPH-dependent reductase of the phosphatidylinositol phosphate (PIP) family—and berberine bridge enzyme-like protein (BBL), have been implicated in the final biosynthetic steps of nicotine and other pyridine alkaloids, but the precise biochemical details remain to be fully elucidated (Deboer et al. 2009; Kajikawa et al. 2009, 2011; Vollheyde et al. 2023).

Genetic manipulation of A622 and BBL enzymes has provided insights into their functions and the potential to engineer tobacco with reduced nicotine content. RNA interference (RNAi)-mediated suppression of *A622* in *Nicotiana* species significantly decreased nicotine and other pyridine alkaloid levels in both hairy root cultures and transgenic plants (Deboer et al. 2009; Kajikawa et al. 2009). Likewise, CRISPR/Cas9-mediated editing of *A622* resulted in a drastic reduction of alkaloid levels, albeit with severely compromised plant growth and development (Burner et al. 2022; Jeong et al. 2024). The genome of *N. tabacum* encodes six *BBL* genes: *BBLa*, *BBLb*, *BBLc*, *BBLd1*, *BBLd2*, and *BBLe* (Kajikawa et al. 2017). RNAi suppression of several *BBL* family members led to a marked decrease in pyridine alkaloid levels (Kajikawa et al. 2011; Lewis et al. 2015). Further investigations involving knocking-out of multiple *BBL* genes revealed that *BBLa*, *BBLb*, and *BBLc* were primarily responsible for nicotine biosynthesis. Interestingly, even with all six *BBL* genes inactivated, tobacco plants still accumulated significant levels of nicotine (Jeong et al. 2024; Lewis et al. 2015, 2020). In contrast, a separate study reported the development of “nicotine-free” tobacco through knocking-out of these six *BBL* genes (Schachtsiek and Stehle 2019). In addition, the inactivation of *BBL* genes also profoundly influenced the level of other pyridine alkaloids, although there are discrepancies in the findings. For example, while some studies reported a concomitant reduction in nornicotine levels (Jeong et al. 2024; Lewis et al. 2015; Schachtsiek and Stehle 2019), others observed an increase (Kajikawa et al. 2011; Lewis et al. 2020). Thus, a comprehensive evaluation of the specific roles of different *BBL* members in the biosynthesis of nicotine and other alkaloids is necessary.

In this study, we first developed a non-transgenic genome editing approach in tobacco by delivering CRISPR/Cas9 with an engineered eggplant mottled dwarf virus (EMDV) vector, a negative-strand RNA virus belonging to the genus *Alphanucleorhabdovirus*, family *Rhabdoviridae* (Walker et al. 2022). Applying this method to the elite *N. tabacum* cultivar Hongda, we successfully generated a large collection of transgene-free, homozygous *BBL* mutants in various combinations within just two generations. Alkaloid profiling of these mutants identified tobacco lines with significantly reduced nicotine content and also provided new insights into alkaloid biosynthesis.

## Results

### Engineering of EMDV for delivering CRISPR/Cas9 nuclease in tobacco plants

**Fig. 1:**
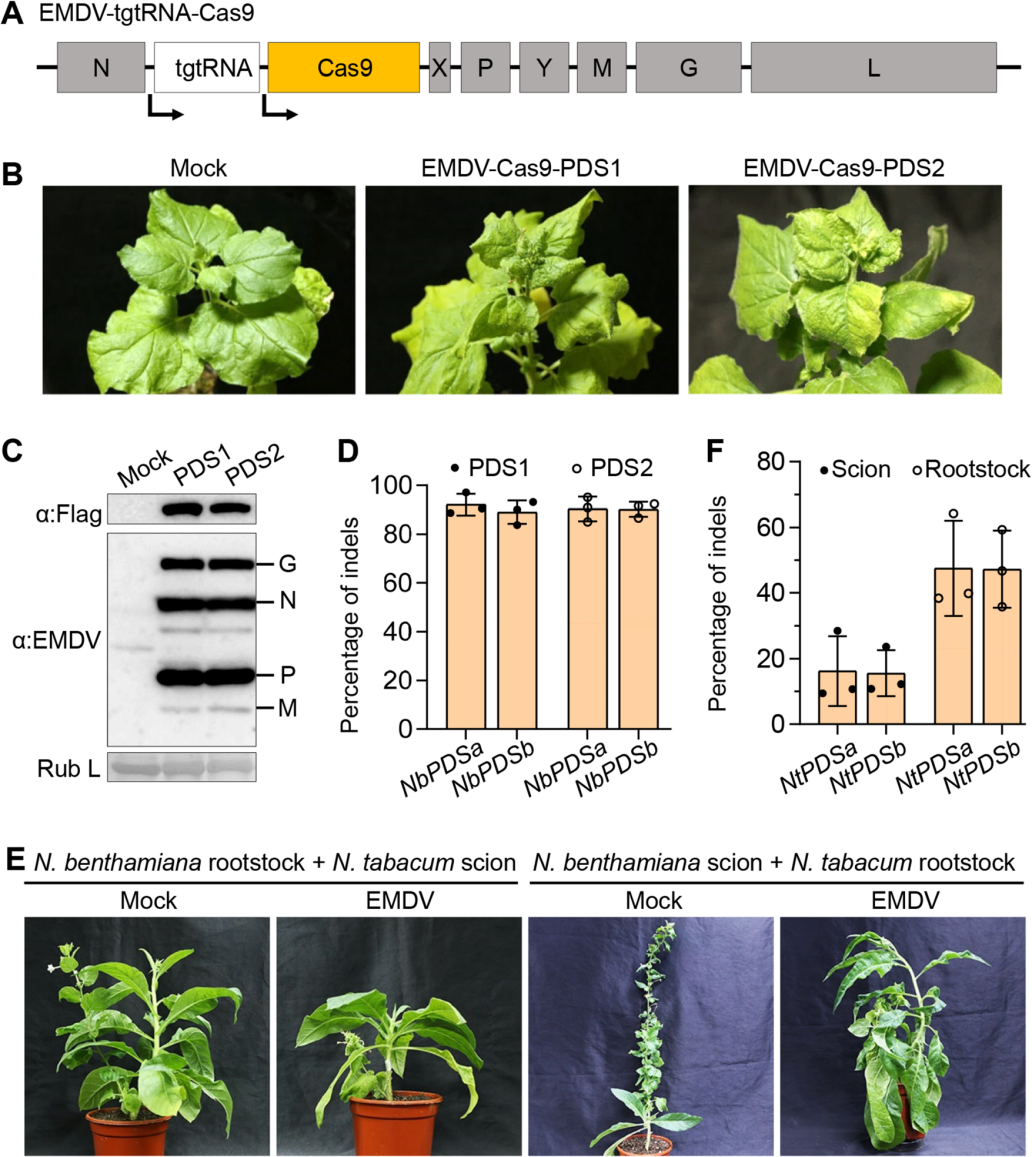
Targeted gene mutagenesis in tobacco plants using the engineered EMDV vector for CRISPR/Cas9 delivery. **A** Schematic representation of the EMDV CRISPR/Cas9 vector. Boxes and lines denote virus coding and non-coding regions, respectively. N, X, P, Y, M, G, and L are viral nucleocapsid protein, a protein of unknown function, phosphoprotein, movement protein, matrix protein, glycoprotein, and large polymerase protein, respectively. Bent arrows indicate viral transcription driven by two duplicated N/X gene-junction sequences. tgtRNA, tRNA^Gly^-sgRNA-tRNA^Gly^ fusion. **B** Symptoms of *N. benthamiana* plants agroinoculated with the EMDV CRISPR/Cas9 vectors targeting the PDS1 and PDS2 sites. Systemically infected plants were photographed at 35 days post-inoculation (dpi). Mock refers to a mock-inoculated plant. **C** Expression of EMDV structural proteins and Cas9 detected by immunoblotting using EMDV-specific antiserum and FLAG antibody. Ponceau S staining of the Rubisco large subunit (Rub L) serves as a protein loading control. **D** Mutagenesis frequency in upper uninoculated leaf tissues of *N. benthamiana* plants infected with the EMDV vectors. Editing efficiencies were determined by HTS of PCR amplicons spanning the PDS1 and PDS2 target sites. **E** Infection of *N. tabacum* plants with the EMDV CRISPR/Cas9 vector through heterograft transmission. *N. tabacum* rootstocks were grated onto *N. benthamiana* scions (left) or vice versa (right), and the *N. benthamiana* parts were mechanically inoculated with the EMDV CRISPR/Cas9 vector containing the PDS2 gRNA. **F** Mutagenesis frequency in leaf tissues of *N. tabacum* plants infected with the EMDV vector. Target-site sequences and mutation types in (**D**) and (**F**) are presented in Table S1. Data are presented as individual data points and mean ± SD for three biological replicates

Based on the recently developed efficient EMDV reverse genetics system (infectious cDNA clone) (Wang et al. 2024), we investigated the feasibility of engineering an EMDV-based vector for CRISPR/Cas9 nuclease delivery in plants. We inserted the coding sequences of *Streptococcus pyogenes* Cas9 and sgRNA into the EMDV genome between the viral *N* and *X* genes as two independent transcription cassettes (Fig. 1A). The expression of both CRISPR elements was individually driven by a duplicated N/X gene-junction sequence containing essential *cis*-elements for viral transcription. Given that nucleorhabdoviruses such as EMDV undergo RNA synthesis in the nucleus (Dietzgen et al. 2021), we flanked the sgRNA with two tRNA^Gly^ precursor sequences to facilitate precise sgRNA processing by recruiting nuclear tRNA-processing enzymes, following a strategy similar to that used in the SYNV-based system (Ma et al. 2020). This engineered vector, designated EMDV-tgtRNA-Cas9, was designed to target separately two sites (PDS1 and PDS2) within the *N. benthamiana Phytoene Desaturase* (*PDS*) gene, which are present in both homoeologs, *NbPDSa* and *NbPDSb*. Upon agroinoculation, the EMDV vectors systemically infected *N. benthamiana* plants, which exhibited slight leaf curling and yellowing symptoms (Fig. 1B). The expression of Cas9 and viral proteins in systemically infected tissues were confirmed (Fig. 1C). For both target sites, high-throughput deep sequencing (HTS) of DNA fragments amplified from virus-infected systemic leaf tissues revealed high mutation frequencies ranging from 89–92% in both *PDS* homoeologs (Fig. 1D). The DNA repair profiles predominantly comprise small deletions of one to eight base pairs (bp) and 1-bp insertion (Fig. S1).

While EMDV can naturally infect several crop species, primarily within the Solanaceae and Cucurbitaceae families, through insect vector transmission (Babaie and Izadpanah 2003; Pappi et al. 2016), we previously observed that mechanical transmission of EMDV was efficient only between *N. benthamiana* plants; however, transmission to other species was more effectively achieved through plant grafting (Wang et al. 2024). To edit the genome of common tobacco (*N. tabacum*), we performed heterografting between *N. benthamiana* and *N. tabacum*. Nearly all grafted plants remained viable, whether *N. benthamiana* served as the rootstock and *N. tabacum* as the scion or vice versa. Next, the *N. benthamiana* grafts were mechanically inoculated with the EMDV-tgtRNA-Cas9 vectors targeting the PDS2 site, which is conserved in *N. tabacum*. Approximately two weeks post-inoculation, the *N. benthamiana* grafts began to display systemic infections, and two weeks later, the viral vector was transmitted to the grafted *N. tabacum*, resulting in evident systemic viral symptoms (Fig. 1E). HTS of amplicons from the *N. tabacum* PDS2 target loci revealed mean mutagenesis frequencies of 16% and 47% when *N. tabacum* was used as the scion and rootstock, respectively (Fig. 1F). The higher indel percentages observed in the latter graft combination (*N. benthamiana* scion + *N. tabacum* rootstock) were likely due to more extensive EMDV infections in the *N. tabacum* grafts.

### Generation and characterization of tobacco *PDS* mutants

**Fig. 2:**
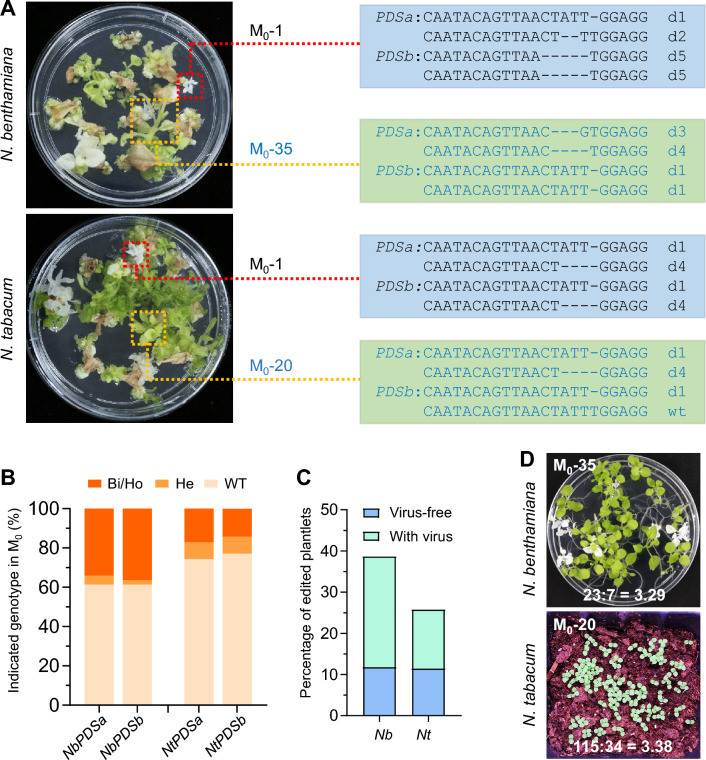
Regeneration of tobacco *PDS* mutants, genotyping, and analysis of progeny.** A** *N. benthamiana* (upper) and *N. tabacum* (lower) plantlets regenerated from leaf explants infected with the EMDV vector. The genotypes of one representative albino and one green plantlet for each plant species are presented. D# denotes the number of base deletions at the PDS2 target site. **B** Bar chart depicting the percentages of regenerated plantlets with each mutation genotype. WT, wild-type; He, heterozygous; Bi, bi-allelic; Ho, homozygous. A total of 44 *N. benthamiana* and 29 *N. tabacum* plantlets were analyzed. **C** Plot illustrating the percentages of virus-free and virus-infected plantlets containing targeted mutations. Nb and Nt represent *N. benthamiana* and *N. tabacum* regenerants, respectively. **D** Phenotypic segregation patterns of tobacco progeny derived from *N. benthamiana* M_0_-35 and *N. tabacum* M_0_-20 lines. The ratios of green to albino progeny are indicated in the images

To generate mutant plants, we collected young symptomatic leaves from the agroinoculated *N. benthamiana* plants (Fig. 1B, right panel) and the *N. tabacum* rootstocks of the grafted plants (Fig. 1E, rightmost panel). The leaf explants were cultured on tissue culture induction medium without antibiotics. Albino plants, indicative of knockouts of all four *PDS* homoeoalleles, were successfully recovered from both *Nicotiana* species (Fig. 2A). Among the M_0_ generation regenerants, 13 out of 44 (29.5%) *N. benthamiana* and 5 out of 35 (14.3%) *N. tabacum* plantlets were albino (Table S1). Genotyping confirmed that these albino plants carried tetra-allelic frameshift mutations. In addition, four plants from each species contained heterozygous or bi-allelic edits at the *PDSa* and *PDSb* loci, while the remaining regenerants were wild-type (Fig. 2A; Table S1). Overall, 38.6% of *N. benthamiana* and 25.7% of *N. tabacum* regenerants contained targeted gene mutations (Fig. 2B). These ratios are considerably lower than the mutation frequencies observed in virus-infected tissues, particularly in *N. benthamiana*, which exhibited ~ 90% somatic mutagenesis (Fig. 1D and F). Reverse transcription-PCR (RT-PCR) analysis of the EMDV vector in *N. benthamiana* regenerants revealed that all wild-type plants were negative for the viral vector, while the majority of edited plants tested virus-positive (Fig. S2). Notably, around 11% of regenerants in both species were virus-free yet contained targeted mutations (Fig. 2C; Table S1), suggesting that the viral vectors were eliminated during tissue culture. These findings indicate that EMDV infections negatively impacted plant regeneration, leading to a disproportionately large number of virus-free, wild-type regenerants.

Two representative green regenerants, *N. benthamiana* M_0_-35 and *N. tabacum* M_0_-20, each containing three frameshift alleles and one non-frameshift or wild-type allele, were selected for self-pollination. The phenotypes of their M_1_ progeny were subsequently analyzed. Consistent with Mendel’s law of segregation, both lines produced albino offspring at an approximate segregation ratio of 3 (green): 1 (albino) (Fig. 2D). All M_1_ progeny were confirmed to be virus-free by RT-PCR analysis.

### EMDV-mediated genome editing of *BBL* genes in* N. tabacum*

**Fig. 3:**
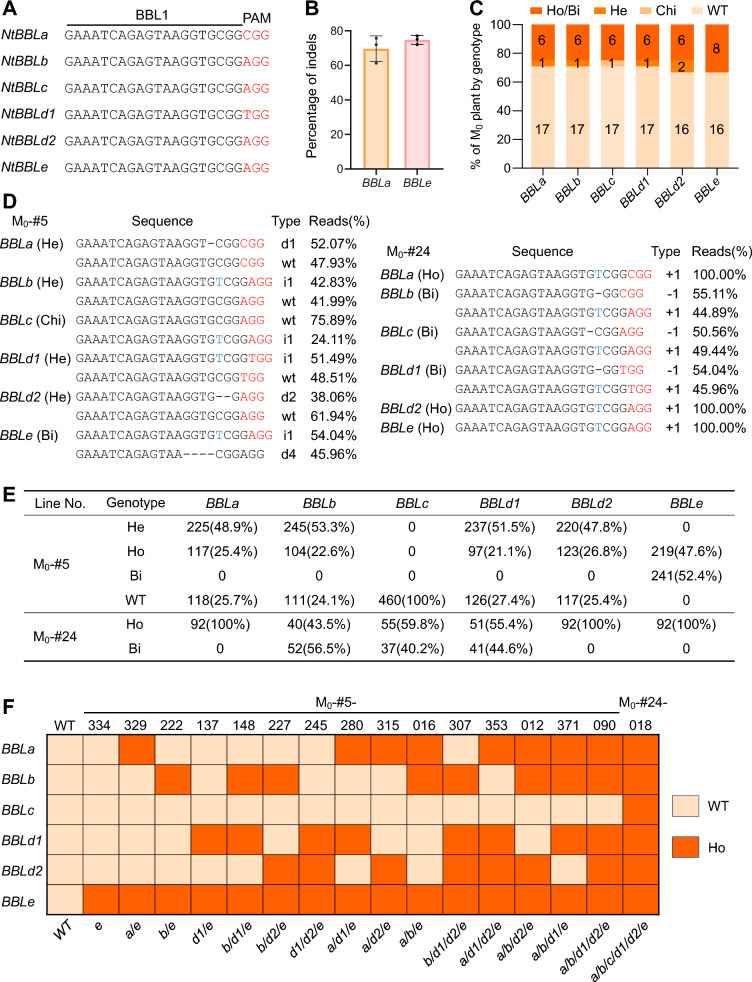
Generation and genotyping of tobacco *BBL* mutants. **A** Sequences of the selected BBL1 site conserved in all six *NtBBL* genes. **B** Mutagenesis frequency in leaf tissues of *N. tabacum* plants infected with the EMDV vector targeting BBL1. Editing efficiencies in *BBLa* and *BBLe* genes were determined by HTS of PCR amplicons spanning the target site.** C** Percentage of regenerated tobacco plantlets with each mutation genotype in the six *BBL* genes. A total of 24 regenerated M_0_ lines were genotyped by HTS. WT, wild-type; Chi, chimeric; He, heterozygous; Bi, bi-allelic; Ho, homozygous. **D** *BBL* target site mutations in M_0_-#5 and -#24 lines. Base deletions (d#) and insertions (i#) are denoted by dashes and blue letters, respectively, with the percentages of reads indicated on the right of each sequence. **E** Summary of the segregation pattern of M_1_ progeny genotypes at the six *BBL* genes. A total of 460 and 92 M_1_ offspring derived from the self-pollinated M_0_-#5 and M_0_-#24 lines were analyzed, and numbers (percentages) of offspring with a particular genotype are shown. **F** schematic representation of a panel of *BBL* mutant lines. Italicized lower case letters indicated particular homozygous *bbl* mutant, e.g., *e* stands for *bble* mutant

Building on the established EMDV delivery approach, we applied this method to edit the *BBL* family genes in *N. tabacum* cv. Hongda. We selected a CRISPR/Cas9 target site (BBL1) that is conserved across all six *BBL* genes (*BBLa*, *BBLb*, *BBLc*, *BBLd1*, *BBLd2*, and *BBLe*) (Fig. 3A) (Steinwand and Ronald 2020). The recombinant EMDV CRISPR/Cas9 vector targeting BBL1 was initially recovered in *N. benthamiana* through agroinoculation and subsequently transmitted to the heterografts of *N. benthamiana* (scion) onto *N. tabacum* (rootstock) via mechanical inoculation. Four weeks post-inoculation, indel frequencies of 69.6% and 74.7% were detected at the *BBLa* and *BBLe* loci, respectively, in the infected tissues of the *N. tabacum* grafts (Fig. 3B). These leaves were then subjected to tissue culture, resulting in the recovery of 24 regenerants, which were genotyped using HTS. The results revealed that 8 (33.3%) regenerants carried targeted mutations, with 6 (25.0%) being homozygous or bi-allelic for frameshift (knockout) mutations across all six *BBL* genes (Fig. 3C; Table S2).

Notably, the M_0_-#5 line exhibited a complex genotype: putatively heterozygous at *BBLa*, *BBLb*, *BBLd1*, and *BBLd2*, bi-allelic at *BBLe*, and chimeric at *BBLc* (Fig. 3D, left panel). We hypothesized that self-pollination of M_0_-#5 would result in M_1_ offspring with various combinations of homozygous *BBL* mutations. To test this, 460 M_1_ segregants were genotyped at all six *BBL* loci using Sanger sequencing (Dataset S1). Genotyping results revealed that while the chimeric mutation in *BBLc* was not inheritable, the heterozygous and bi-allelic mutations in the other five *BBL* genes were successfully transmitted to the M_1_ offspring. This generated a collection of 36 mutant lines with homozygous mutations in one to five *BBL* loci, corresponding to 15 unique genotypes (Fig. 3F; Table S3). Further analysis of the genotypes of M_1_ progeny in each *BBL* locus revealed segregation ratios of 1 (homozygote): 2 (heterozygote): 1 (wild-type) for the heterozygous mutations in *BBLa*, *BBLb*, *BBLd1*, and *BBLd2* and a ratio of approximately 1 (homozygote): 1 (bi-allelic) for the bi-allelic mutations in *BBLe* (Fig. 3E). Similarly, 92 offspring derived from M_0_-#24, which contained bi-allelic or homozygous mutations across all six *BBL* loci, were also genotyped (Fig. 3D, right panel). Again, the data confirmed stable transmission of the mutations to the M_1_ generation following classical Mendelian inheritance (Fig. 3E). From these M_1_ offspring, we selected a panel of 16 non-redundant mutant lines containing single, double, or high-order homozygous mutations across the six *BBL* genes (Fig. 3F). These plants were self-pollinated to produce M_2_ offspring for subsequent analysis.

### Pyridine alkaloid profiling of tobacco *BBL* mutant lines

**Fig. 4:**
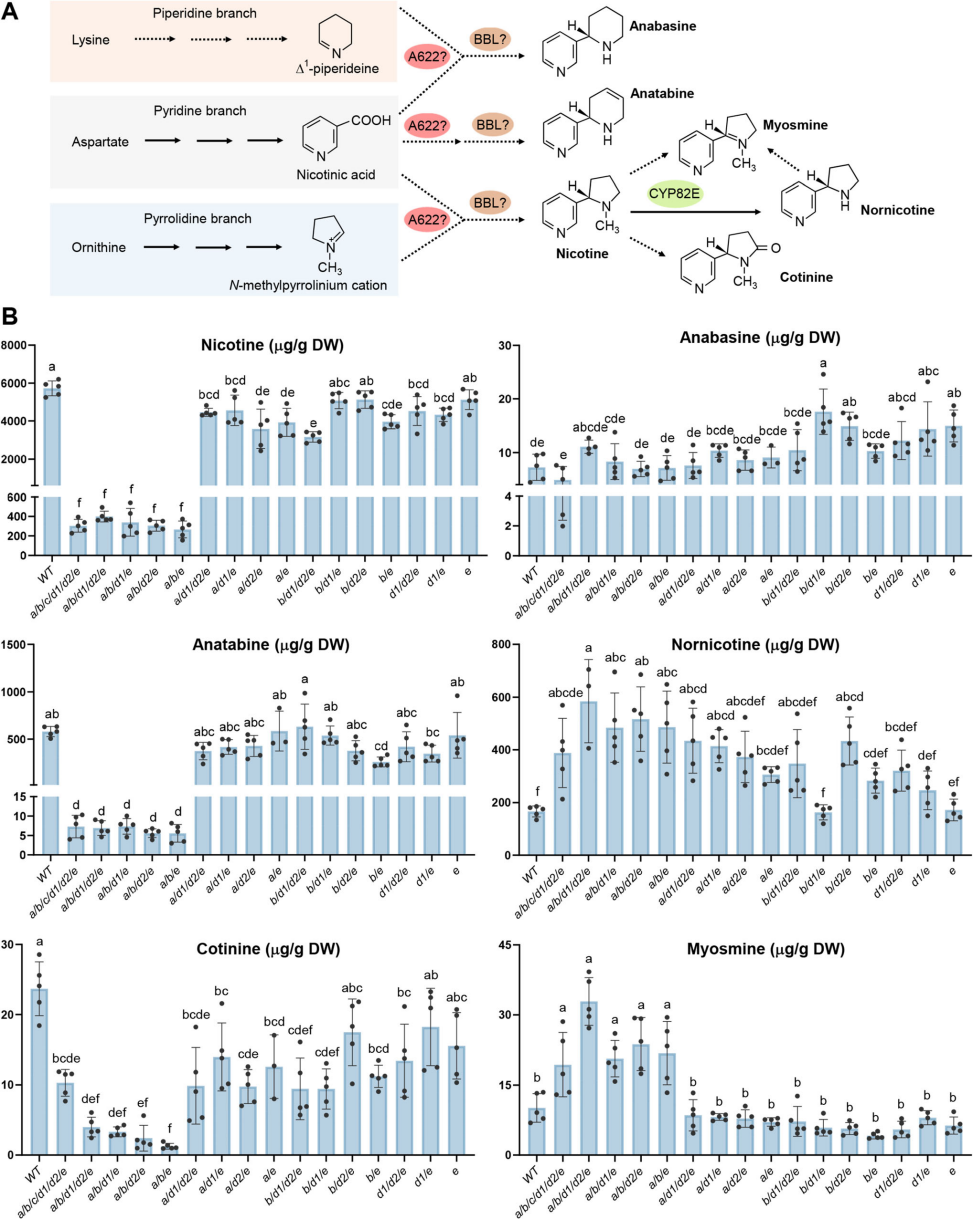
Analysis of pyridine alkaloid content of *N. tabacum BBL* mutant lines. **A** Schematic diagram of pyridine alkaloid biosynthesis in tobacco. Full arrows represent characterized enzymatic steps, and dashed arrows represent undefined or hypothetic steps. Question marks next to the enzymes signify that their precise catalytic reactions have not been confirmed by biochemical assays. A622, PIP family reductase; BBL, berberine bridge enzyme-like protein; CYP82E, cytochrome P450 monooxygenase. The figure is adapted from Kaminski et al. (2020) and Vollheyde et al. (2023) with modifications. **B** Comparison of alkaloid content in leaves of tobacco (*N. tabacum* cv. Honda) wild-type (WT) and *BBL* mutants. Leaf samples were collected at the flower–bud stage, and the levels of alkaloids, expressed as μg/g dry leaf weight (DW), were measured using GC–MS. *BBL* genotypes are shown below the bars. Data are presented as individual data points and mean ± SD. Different letters above the bars indicate statistically significant differences determined using one-way ANOVA followed by *post-hoc* Tukey tests. Means that do not share a letter in common are different at the *P* < 0.05 level of significance

The BBL enzymes are proposed to catalyze the coupling reactions of nicotinic acid or its metabolites with *N*-methylpyrrolinium cation, ∆^1^-piperidine, and a nicotinic acid derivative to synthesize nicotine, anabasine, and anatabine, respectively (Fig. 4A). In tobacco, nicotine can be further metabolized into other alkaloids, including nornicotine, myosmine, and cotinine (Fig. 4A) (Kaminski et al. 2020).

The 16 *BBL* mutant lines, along with a wild-type control, were cultivated in a greenhouse, and the pyridine alkaloid content in leaves of non-topped plants was quantified using gas chromatography-mass spectrometry (GC–MS) (Fig. 4B). Based on nicotine levels, the mutant lines were categorized into two groups. The first group, comprising the line with homozygous *bbla, bblb,* and *bble* mutants (abbreviated *a/b/e*) and its high-order mutants* a*/*b*/*d1*/*e*, *a*/*b*/*d2*/*e*, *a/b/d1/d2/e*, and* a*/*b*/*c*/*d1*/*d2*/*e*, exhibited significantly reduced nicotine levels ranging from 0.27 to 0.40 mg/g, corresponding to a 14.4 - to 21.6 - fold reduction compared to the wild-type. The second group included the remaining lines, which showed only slight to modest reductions in nicotine content relative to the wild-type.

The lack of significant differences in nicotine content among the five lines in the first group suggests that introducing the *BBLc*, *BBLd1*, or *BBLd2* mutations into the *a/b/e* genetic background did not further decrease nicotine levels. Furthermore, comparisons of various *BBL* genotypes between the two groups with disparate nicotine levels, such as the low-nicotine genotype *a/b/e* versus the high-nicotine genotypes *a/e* and *b/e*, and similar comparisons of *a*/*b*/*d1*/*e* versus *a*/*d1*/*e* and *b*/*d1*/*e*, *a*/*b*/*d2*/*e* versus *a*/*d2*/*e* and *b*/*d2*/*e*, *a/b/d1/d2/e* versus *a/d1/d2/e* and *b/d1/d2/e*, revealed that the presence of *BBLa* or *BBLb* in any mutant background is sufficient to sustain a high nicotine level. Given that *BBLe* mutations were present in all lines with near-wild-type levels of nicotine, it is likely that this gene is not essential for nicotine biosynthesis. Overall, these findings suggest that *BBLa* or *BBLb* play a pivotal role in nicotine biosynthesis in *N. tabacum* cv. Hongda.

Profiling of other pyridine alkaloids revealed a positive correlation between nicotine and anatabine levels. The five mutant lines in the low-nicotine group also exhibited significantly reduced anatabine content (Fig. 4B). Cotinine, a minor tobacco alkaloid potentially derived from chemical oxidation of nicotine, followed a similar trend, although the observed differences were more variable. This variability likely stems from the low concentrations of cotinine, which can complicate precise measurement. In contrast, anabasine levels remained largely unaffected across most of the combinatory mutant lines, indicating that *BBL* enzymes are dispensable for the condensation of piperidine and pyridine rings. Notably, nornicotine levels, an alkaloid thought to arise from the oxidative *N*-demethylation of nicotine, were elevated in most of the mutant lines. This increase was particularly pronounced in the five low-nicotine lines, which showed a 2.3 - to 3.5 - fold increase compared to the wild-type. In addition, these lines also exhibited a 1.9 - to 3.2 - fold increase in myosmine levels, a pyridine alkaloid hypothesized to result from nornicotine degradation.

### Phenotypic evaluations of tobacco *BBL* mutant lines

**Fig. 5:**
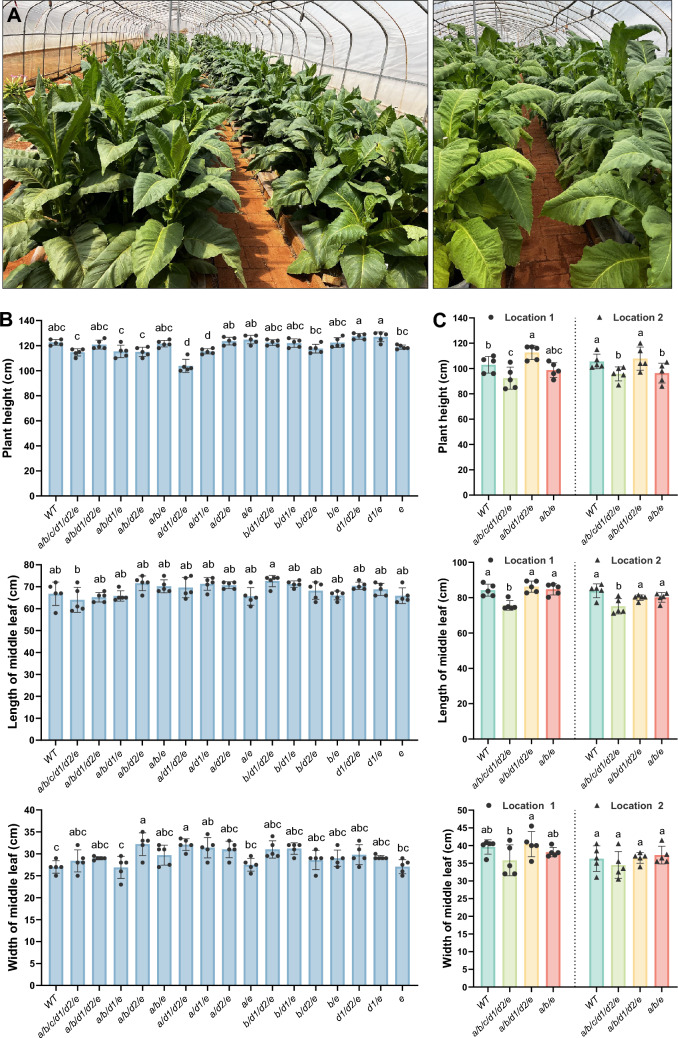
Growth phenotype of tobacco *BBL* mutants. **A** Images showing tobacco wild-type and indicated *BBL* mutant plants before topping (leaf) and after topping (right) grown in a greenhouse. Plants were photographed at 98 and 140 days after seed sowing. Five wild-type plants are located at the far end of the right row. **B**, **C** Bar charts showing plant height (upper), middle leaf length (middle), and width (bottom) of wild-type and *BBL* mutant lines grown under (**B**) Greenhouse conditions or (**C**) Field conditions at two different locations. Plant-growth parameters were measured at (**B**) Forty-five days and (**C**) Ten days after topping. Data are presented as individual data points and mean ± SD. Different letters above the bars indicate statistically significant differences determined using one-way ANOVA followed by *post-hoc* Tukey tests (*P* < 0.05)

Under greenhouse conditions, all mutant lines exhibited normal growth and development (Fig. 5A). However, measurement of mean plant height revealed that several high-order mutants, such as *a*/*b*/*c*/*d1*/*d2*/*e*,* a*/*b*/*d1*/*e*,* a*/*b*/*d2*/*e*,* a*/*d1*/*d2*/*e*, and *a*/*d1*/*e*, were approximately 6% to 12% shorter than the wild-type plants. Whereas the *a*/*b*/*d2*/*e* and* a*/*d1*/*d2*/*e* lines displayed an increased middle leaf width, the middle leaf length across all lines remained comparable to that of the wild-type plants (Fig. 5B).

In addition, three low-nicotine mutant lines, namely *a*/*b*/*c*/*d1*/*d2*/*e*,* a*/*b*/*d1*/*d2*/*e*, and* a*/*b*/*e*, were selected for growth phenotype evaluation in two field locations. The *a*/*b*/*c*/*d1*/*d2*/*e* line demonstrated a roughly 10% reduction in plant height and a 9% reduction in middle leaf length compared to the wild-type plants. In contrast, the *a*/*b*/*d1*/*d2*/*e* and* a*/*b*/*e* genotypes did not exhibit statistically significant differences in plant height, middle leaf length, or middle leaf width when compared to wild-type (Fig. 5C).

## Discussion

Building on our prior success with transformation-free genome editing using CRISPR/Cas9 delivered by SYNV (Ma et al. 2020), a model rhabdovirus that infects *N. benthamiana* but not *N. tabacum*, we employ EMDV to overcome the host range limitations. Both SYNV and EMDV are nucleorhabdoviruses that undergo nuclear replication, a unique characteristic among known plant RNA viruses (Dietzgen et al. 2021). Leveraging this property, we adopted a tRNA-processing strategy to ensure the precise release of sgRNA from viral RNA transcripts, addressing a technical challenge inherent to RNA virus-mediated sgRNA expression that remained unresolved in most cases. This strategy, previously demonstrated to be effective in SYNV-based vectors (Ma et al. 2020), also resulted in efficient somatic editing induced by EMDV-based CRISPR vectors. These findings highlight the large carrying capacity of plant rhabdovirus vectors in general and their utility in delivering the entire CRISPR/Cas9 machinery.

Mutations induced in EMDV-infected somatic tissues were integrated into germline cells through tissue culture, resulting in 25.7% (*PDS*) and 33.3% (*BBL*) of *N. tabacum* regenerants carrying heritable edits. While these ratios are comparable to those achieved with TSWV-based CRISPR vectors in tobacco (Liu et al. 2023), they are considered relatively low given the high frequencies of somatic mutagenesis. The study by Liu et al. (2023) demonstrated that antiviral treatments during tissue culture effectively eliminated TSWV vector and promoted plant regeneration from edited cells. Implementing similar strategies in the EMDV system might also mitigate the negative impact of EMDV infection on plant regeneration, thereby enhancing the recovery of regenerants with targeted mutations. Nevertheless, with the current method we found that a significant proportion of the edited *BBL* regenerants (six out of eight) were sextuple knockout mutants, and virus-free, homozygous mutants were readily obtained in the M_1_ generation, underscoring the effectiveness of the EMDV-based CRISPR/Cas9 delivery system.

Through the utilization of a serendipitous regenerant (M_0_-#5) heterozygous for mutations in multiple *BBL* loci, we successfully obtained 15 mutant lines with various combinations of *BBL* mutants from its self-progeny. This approach, involving the use of sexual segregants, minimizes the potential somatic variations that can arise from tissue culture, thereby ensuring greater genetic uniformity in the resulting lines. Alkaloid profiling of these mutant lines, along with a homozygous sextuple mutant, revealed that BBLa and BBLb are the primary BBL enzymes involved in nicotine biosynthesis, while the other BBL members play only minor roles. These findings align with previous gene expression analyses that identified *BBLa* and *BBLb* as the most highly expressed members in tobacco (Kajikawa et al. 2017), as well as with alkaloid analyses of *BBL* mutants in the K326 and TN90 tobacco cultivars (Lewis et al. 2015, 2020).

The reduction, but not complete elimination, of nicotine content in our low-nicotine lines, ranging from 14.4- to 21.6-fold, is consistent with the reductions (up to 17-fold) reported by Jeong et al. (2024) and Lewis et al. (2020). The presence of appreciable, though significantly reduced, amounts of nicotine even in the sextuple *BBL* knockout mutants suggests that nicotine may also be synthesized via an uncharacterized route independent of BBL enzymatic activities (Lewis et al. 2020). A recent study by Vollheyde et al. (2023), employing genetic *BBL* mutants, chiral analysis, and precursor feeding experiments, provided new insights into nicotine biosynthesis. The study found that while wild-type *N. benthamiana* plants accumulated only (*S*)-nicotine, the residual nicotine present in mutant lines lacking any functional BBL existed as racemic mixtures. This led to the hypothesis that the residual nicotine in *BBL* mutant lines arises from a spontaneous reaction, whereas in wild-type plants the presence of BBL enzymes accelerate an enantioselective reaction, producing exclusively (*S*)-nicotine (Vollheyde et al. 2023). In wild-type tobacco plants, although the (*S*)-enantiomer accounting for ∼99.8% of total nicotine accumulated in cured leaves (Armstrong et al. 1998), 4% of nicotine exist as (*R*)- enantiomer at the point of synthesis in the root (Cai et al. 2013). This proportion of (*R*)-nicotine at synthesis corresponds with the residual nicotine content (4.6 − 6.9%) observed in the low-nicotine mutant lines.

Another notable finding in our study is the significant increase in nornicotine accumulation observed in all five low-nicotine mutant lines, although several high-nicotine lines also exhibited an increase and the data values showed considerable variability among individual plant samples. Elevated nornicotine levels have previously been observed in the roots of tobacco *BBL* RNAi lines (Kajikawa et al. 2011; Lewis et al. 2015) and in the leaves of *BBL* knockout lines (Lewis et al. 2020). Supporting the accumulation of high nornicotine levels in these low-nicotine lines is the concomitant significant increase in myosmine levels. Myosmine, a minor tobacco alkaloid, is thought to arise from nornicotine degradation, and its levels have consistently been found to positively correlate with nornicotine levels across all *Nicotiana* species analyzed (Kaminski et al. 2020).

Nornicotine is produced by *N*-demethylation of nicotine, a reaction catalyzed by the CYP82E subfamily of cytochrome P450 monooxygenases (Lewis et al. 2008, 2010; Siminszky et al. 2005). Thus, the observed increase in nornicotine content in mutant lines with dramatically reduced nicotine levels seems counterintuitive. However, it is important to note that neither our study nor the studies mentioned above discriminated between the two enantiomers of nornicotine. Unlike the low and consistent percentage (~ 0.2%) of the (*R*)-nicotine accumulated in tobacco leaves, the fraction of (*R*)-nornicotine is considerably elevated and highly variable, ranging from 4 to 75% depending on various conditions (Cai et al. 2012). This high variation arises from several factors, including the relative abundances, spatial–temporal expression patterns, and enantioselective properties of the three CYP82E enzymes in tobacco (Cai & Bush 2012; Cai et al. 2013). It is conceivable that the absence of BBL enzymes may lead to an elevated level of (*R*)-nicotine produced via the spontaneous reaction proposed by Vollheyde et al. (2023). This (*R*)-nicotine is subsequently converted to (*R*)-nornicotine by the highly enantioselective demethylases CYP82E5v2 and CYP82E10 present in root tissues (Cai et al. 2012), thereby contributing to the increased nornicotine levels observed in the leaves. Given that nornicotine can be easily converted into the* N*′-nitrosonornicotine, a well-characterized carcinogen, during the curing and processing of tobacco, reducing nornicotine levels has been a significant breeding goal (Lewis et al. 2008). Together, these findings underscore the necessity of targeting additional gene families within the nicotine biosynthetic pathway to further reduce nicotine content and simultaneously lower nornicotine levels in tobacco, as exemplified in a recent study by Kernodle et al. (2022).

We conducted a preliminary evaluation of the growth phenotypes of the *BBL* mutant collection in a greenhouse and a small-scale open field evaluation of three low-nicotine mutant lines. Based on the limited agronomic trait data collected, the *a*/*b*/*c*/*d1*/*d2*/*e* genotype exhibited significantly reduced growth, consistent with the phenotypes observed in similar sextuple mutants of other tobacco cultivars (Lewis et al. 2015, 2020). However, two other genotypes, *a*/*b*/*d1*/*d2*/*e* and *a*/*b*/*e*, showed promising results as they performed similarly to wild-type controls. To fully assess the agronomic potential of these mutants, more rigorous evaluations, including cured leaf yield and quality, are needed in planned future studies.

## Materials and methods

### Plant materials and growth conditions

*N. benthamiana* lab strain and *N. tabacum* flue-cured cultivars K326 and Hongda were cultivated in controlled growth chambers maintained at 25 ± 2 °C, with a 16-h light/8-h dark photoperiod, 60% relative humidity, and a photosynthetic photon flux density (PPFD) of *ca*. 150 μmol m^−2^ s^−1^. For alkaloid content analysis and growth phenotype assessment, *N. tabacum* cv. Hongda wild-type and *BBL* mutant lines were grown in an insect-proof greenhouse at the Yunnan Academy of Tobacco Science research station, as well as in two open field locations in Kunming, China.

### Construction of EMDV CRISPR/Cas9 vectors

The binary construct containing the full-length EMDV cDNA clone (pEMDV( +)), along with the supporting plasmids pGD-N, pGD-P, and pCB-L, which were designed to express EMDV core proteins necessary for recovery of recombinant viruses, were described earlier by Wang et al. (2024). To engineer EMDV vectors for the expression of sgRNA and Cas9, and to facilitate the insertion of target site-specific spacer sequence, we first constructed an intermediate vector, termed pGD Bsp-AarI-Xma. This vector contains an EMDV cDNA fragment spanning from the *Bsp*119 I site to *Xma* J site, a tgtRNA transcription cassette with its spacer sequence replaced by two *Aar* I restriction sites, and a Cas9 transcription cassette. For this purpose, three EMDV-derived fragments we amplified by PCR using pEMDV( +) as the template and the following primer pairs: pGD/Bsp119 I/F and NXJ/I/R, NXJ/II/F and NXJ/IIR, NXJ/IIIF and pGD/XmaJ I/R. In addition, three CRISPR fragments were amplified from pSYNV-tgtRNA-Cas9 (Ma et al. 2020) using the primer pairs sgRNA/F and sgRNA/AarI/R, sgRNA/AarI/F and sgRNA/R, and Cas9/F and Cas9/R. The six fragments, each containing approximately 15-nt terminal overlapping sequences, were assembled with the linearized pGD vector (Goodin et al. 2002) using In-Fusion HD cloning reagents (Takara, China), generating pGD Bsp-AarI-Xma.

To generate EMDV vectors with a custom sgRNA targeting a specific DNA locus, two partially complementary oligonucleotides corresponding to the selected spacer sequence were synthesized, annealed, and inserted into the pGD Bsp-AarI-Xma plasmid via *Aar* I Golden Gate cloning. Inserts assembled in this intermediate vector were then sub-cloned into pEMDV( +) through Bsp119 I and XmaJ I double digestion to generate the pEMDV-tgtRNA-Cas9 vectors. The sequences of oligonucleotides used for cloning are provided in Table S4.

### Inoculation of EMDV vectors

Agroinoculation, mechanical inoculation, and heterograft transmission of EMDV vectors were performed following previously established protocols (Wang et al. 2024). Briefly, the pEMDV-tgtRNA-Cas9, pGD-N, pGD-P, and pCB-L constructs, along with three binary plasmids coding for viral suppressors of RNA silencing (VSRs) (pGD-P19, pGD-γb, and pGD-HcPro) (Ganesan et al. 2013; Wang et al. 2015) were separately mobilized into *Agrobacterium tumefaciens* strain EHA105 by electroporation. The bacterial strains harboring these plasmids were adjusted at a final optical density (OD_600_) of 0.08, except for the strain carrying pEMDV-tgtRNA-Cas9, which was set at OD_600_ of 0.23. The bacterial suspensions were then mixed and infiltrated into the leaves of 4 ~ 5-week-old *N. benthamiana* plants.

For heterografting, 6 ~ 8-week-old *N. benthamiana* and *N. tabacum* seedlings were used, and wedge grafting was performed according to the protocol previously described (Notaguchi et al. 2020). The grafted plants were placed in a growth chamber maintained at 27 °C and 80% relative humidity with a 24-h light photoperiod for 7 days, after which they were transferred to a standard growth chamber.

For mechanical inoculation of the grated plants, 1 ~ 2 g of symptomatic leaf tissues from *N. benthamiana* plants infected EMDV vectors were collected and ground in a mortar with 3 to 5 mL of Paul’s buffer [5-mM diethyldithiocarbamate (DIECA), 1 mM EDTA, 5 mM sodium thioglycolate in 0.05 M potassium phosphate buffer (pH 7.0), 2% Celite and a spoonful of activated charcoal]. The resulting leaf sap was gently rubbed onto two leaves of the grafted *N. benthamiana* plants. After inoculation, the plants were immediately rinsed with tap water and kept in a growth chamber.

### Generation of genome-edited plants

Mutant plant regeneration via tissue culture was conducted as previously described (Ma et al. 2020). Approximately 1-week post-systemic EMDV infection, upper infected leaves from *N. benthamiana* and *N. tabacum* plants were harvested and used as explants for tissue culture. The leaves were sterilized using 70% ethanol followed by 0.1% mercuric chloride, cut into discs, and then cultured on Murashige and Skoog [MS] agar medium without any antibiotics and with 1 mg/L 6-benzylaminopurine (6-BA), for 4 to 6 weeks. The regenerated shoots were excised, transferred to non-selective MS rooting medium devoid of 6-BA, and cultured for an additional 20 days. The rooted plantlets (5–6 cm in length) were acclimated and then transplanted into soil.

### Analysis of mutation frequencies and genotyping

Deep sequencing was employed to analyze somatic mutagenesis frequency in virus-infected *N. benthamiana* and *N. tabacum* plants. Genomic DNA was extracted from young, symptomatic upper leaf tissues. The target site fragments were amplified through a first-round PCR using Phusion High-Fidelity DNA Polymerase (NEB) with locus-specific primers containing 5′-appended Illumina adapter sequences (Table S5). Subsequently, a second-round PCR was performed for barcoding, using Illumina index primer pairs that anneal to the adapter sequences. The barcoded amplicon libraries were quantified, diluted to a concentration of 1 ng/µL, and sequenced using the Illumina HiSeq platform. Paired-end reads were pre-processed and aligned to a reference sequence using the Hi-Tom online platform (http://www.hi-tom.net/hi-tom/), as previously described (Liu et al. 2019b). Mutagenesis frequency was calculated by dividing the number of reads with indels by the total number of sequenced reads.

For the genotyping of the regenerated M_0_ lines, deep sequencing was conducted using the above-mentioned procedure. Putative genotypes were assigned according to the following criteria, as previously described (Li et al. 2021; Liu et al. 2023): WT, 0% ≤ indels% ≤ 10.0%; Chimeric, 10% < indels% ≤ 35.0%; Heterozygous, 35.0% < indels% ≤ 80.0%; Homozygous, 80.0% < indels% for one mutation type ≤ 100.0%; Biallelic, 80.0% < indels% for two mutation types ≤ 100.0%.

M_1_ and M_2_ progeny were genotyped by Sanger sequencing. Target DNA regions were amplified by PCR using locus-specific primers (Table S5) in a high-throughput format and subsequently Sanger sequenced (Higentec, Changsha, China). For bi-allelic and heterozygous mutations with superimposed sequencing chromatograms, deconvolution of sequences was performed using the online tool ICE (https://ice.synthego.com/#/).

### T7 endonuclease 1 (T7E1) assay

To assess the presence of mutations at the *NbPDSa* and *NbPDSb*, DNA fragments spanning the target site were amplified from the *N. benthamiana* regenerants using specific primer sets listed in Table S5. The PCR products were gel isolated and quantified, and equal quantities of each PCR products were mixed with DNA extracted from wild-type plants. The DNA mixtures were then denatured and renatured in NEB buffer 2 (50 mM NaCl, 10 mM Tris–HCl, 10 mM MgCl_2_, 1 mM DTT, pH 7.9) under the following thermal cycling conditions: 95 °C for 5 min, ramping down from 95 °C to 85 °C at -2 °C/sec, further ramping down from 85 °C to 25 °C at -0.1 °C/sec, and finally holding at 4 °C for 5 min. After annealing, the DNA samples were digested with 5 U of T7EI (NEB, China) at 37 °C for 15 min. The digested DNA was analyzed by agarose gel electrophoresis to detect cleavage indicative of mutations.

### Immunoblotting

For protein analysis, total proteins were extracted from the leaf samples, separated by 12.5% SDS-PAGE, and transferred to nitrocellulose membranes. The membranes were probed with a polyclonal antiserum raised against EMDV virions (1:5000; DSMZ, Germany) or monoclonal antibodies against FLAG epitope (Sigma-Aldrich, MO, USA), and then with goat anti-mouse or anti-rabbit secondary antibodies (1:10,000; HuaAn, China). Protein bands on the blots were visualized using Enhanced Chemiluminescence (ECL) Western Blotting Detection Reagents (Fdbio Science, Hangzhou, China).

### RT-PCR

Total RNAs were extracted from the leaf tissues of regenerated plants using Trizol reagent (Invitrogen Life Technologies). Reverse transcription was performed using the ReverTra Ace qPCR RT Master Mix and gDNA Remover kit (Toyobo), ensuring removal of genomic DNA. The resulting cDNA was then used as a template for PCR amplification with primers specific to the EMDV *N* gene and the *N. benthamiana GAPDH* genes (Table S4).

### Tobacco alkaloid profiling

Tobacco wild-type and *BBL* mutant lines were cultivated in soil in a greenhouse. Five M_2_-offspring plants for each genome-edited line were grown, and their genotypes were confirmed by Sanger sequencing. At the flower–bud stage, six leaf disks (three per leaves) without midvein were collected from the 9th to 10th leaf positions of each plant and pooled into a single tube. The leaf samples were cryodesiccated and ground into a fine powder. A 0.2-g portion of the leaf powder was thoroughly mixed with 2 mL of 5% (w/v) sodium hydroxide aqueous solution, followed by the addition of 50 µL of an internal standard solution containing 1.0 mg/mL dimethyl quinoline and 0.5 mg/mL 2,3-bipyridine dissolved in methanol and diluted with dichloromethane. Metabolites were extracted by vortexing with 10 mL of an extracting solution (dichloromethane: methanol (v/v) = 4:1) at 2,000 rpm for 40 min, followed by a 1-h incubation at room temperature. The samples were then centrifuged at 12,000 rpm for 1 min, and the organic phase was collected for alkaloid profiling.

Alkaloid content was analyzed using a GC–MS system (TQ/SQ, Bruker, Bremen, Germany) equipped with a DB-35MS column (30 m × 0.25 mm × 0.25 µm) (Agilent, Santa Clara, CA, USA). The compounds were separated under a helium gas flow rate of 1 mL/min, with an inlet temperature set at 250 °C. The split ratios were 40:1 for nicotine and 10:1 for other alkaloids. The oven temperature program began with an initial hold at 100 °C for 3 min, then ramped up to 260 °C at a rate of 8 °C/min, and was held at 260 °C for 10 min. The transfer line and ion source temperatures were maintained at 280 °C and 250 °C, respectively. The ionization mode used was electron impact at 70 eV, with a solvent delay of 5 min for nicotine and 7 min for the other alkaloids. Detection was performed using a SIM detector, and data acquisition was performed with Compass CDS software.

## Supplementary Information

Below is the link to the electronic supplementary material.

Supplementary file1 (XLSX 70 KB)

Supplementary file2 (PDF 695 KB)

## Data Availability

The data that support the findings of this study are available in the article or online Supplementary Information, or from the corresponding author upon reasonable request.
