# Supplementary material for: Development of an RNA virus vector for non-transgenic genome editing in tobacco and generation of berberine bridge enzyme-like mutants with reduced nicotine content
Source: aBIOTECH. 2024 Nov 22;5(4):449–64. doi: 10.1007/s42994-024-00188-y (PMC11624166; doi:10.1007/s42994-024-00188-y)
Supplement: Supplementary file 2 — Supplementary file2 (PDF 695 KB) [file 42994_2024_188_MOESM2_ESM.pdf]

Development of an RNA virus vector for non-transgenic genome editing in tobacco and generation of *Berberine Bridge Enzyme-Like* mutants with reduced nicotine content

Haiying Xiang[1,#], Binhuan Chen[2,3,4,#], Shuo Wang[2,3,4,#], Wanli Zeng[1], Jiarui Jiang[1], Jianduo Zhang[1], Li Xu[1], Shuang Ni[2,3,4], Qian Gao[1*], Zhenghe Li[2,3,4*]

[1]Yunnan Academy of Tobacco Science, Kunming, Yunnan, 650106, China.

[2]State Key Laboratory of Rice Biology, Institute of Biotechnology, Zhejiang University, Hangzhou, China

[3]Ministry of Agriculture Key Laboratory of Molecular Biology of Crop Pathogens and Insect Pests, Zhejiang University, Hangzhou, China

[4]Key Laboratory of Biology of Crop Pathogens and Insects of Zhejiang Province, Zhejiang University, Hangzhou, China

[#]These authors contributed equally to this work.

*Correspondence Authors: Zhenghe Li (lizh@zju.edu.cn); Qian Gao (gaoqian840905@163.com)

Supplementary Information

Table of Contents

- **Supplementary Figures**

  Fig S1 Somatic mutation types and frequencies in *N. benthamiana* and *N. tabacum* plant tissues infected with EMDV-tgtRNA-Cas9 vectors

  Fig S2 Detection of target site mutations and the presence of EMDV vector in regenerated N. benthamiana plants

- **Supplementary Tables**

  Table S1 Summary of the phenotypes and *PDS* genotypes of *N. benthamiana* and *N. tabacum* M_0 lines regenerated from virus-infected tissues

  Table S2 Summary of the *BBL* genotypes of *N. tabacum* M_0 lines regenerated from virus-infected tissues

  Table S3 Summary of the *BBL* genotypes of *N. tabacum* M1 lines derived from M_0-#5 and M_0-#24

  Table S4 List of primers used in plasmid construction and RT-PCR

  Table S5 List of oligos used for HTS, Sanger sequencing, and T7E1 assay

Target site: PDS1

```
TTGGTAGTAGCGACTCCATGGGG  (Reference)
```

NbPDSa (Niben101Scf01283)

```
TTGGTAGTAGCGACTCCATGGGG   WT   11.82%
TTGGTAGTAGCGA---CATGGGG   d3   37.29%
TTGGTAGTAGCGACTCCCATGGGG  i1   20.31%
TTGGTAGTAGCGACTC-ATGGGG   d1   18.44%
TTGGTAGTAGC------ATGGGG   d6   12.41%
```

NbPDSb (Niben101Scf14708)

```
TTGGTAGTAGCGACTCCATGGGG   WT   11.30%
TTGGTAGTAGCGA---CATGGGG   d3   23.62%
TTGGTAGTAGCGACTC-ATGGGG   d1   18.57%
TTGGTAGTAGCGACTCCCATGGGG  i1   15.38%
TTGGTAGTAGCGA----ATGGGG   d4   11.54%
TTGGTAGTAG------CATGGGG   d6   11.06%
TTGGTAGTAG-------ATGGGG   d7   8.53 %
```

Target site: PDS2

```
CAATACAGTTAACTATTTGGAGG  (Reference)
```

NbPDSa (Niben101Scf01283)

```
CAATACAGTTAACTATTTGGAGG   WT   6.74%
CAATACAGTTAACTATT-GGAGG   d1   41.47%
CAATACAGTT--------GGAGG   d8   20.23%
CAATACAGTTAACTAT--GGAGG   d2   16.32%
CAATACAGTTA------TGGAGG   d6   8.00%
CAATACAGTTAACT----GGAGG   d4   7.24%
CAATACAGT---------GGAGG   d9   6.74%
```

NbPDSb (Niben101Scf14708)

```
CAATACAGTTAACTATTTGGAGG   wt   11.34%
CAATACAGTTAACTATT-GGAGG   d1   52.39%
CAATACAGTTA------TGGAGG   d6   12.59%
CAATACAGTTAACT----GGAGG   d4   11.84%
CAATACAGTTAACT---TGGAGG   d3   11.84%
```

NtPDSa (Nitab4.5 0004950)

```
CAATACAGTTAACTATTTGGAGG   wt   36.63%
CAATACAGTTAACTATT-GGAGG   d1   47.01%
CAATACAGTTAACTAT--GGAGG   d2   6.80%
CAATACAGTTAACTATTTTGGAGG  i1   5.57%
CAATACAGTTAACT----GGAGG   d4   3.99%
```

NtPDSb (Nitab4.5 0006338)

```
CAATACAGTTAACTATTTGGAGG   wt   41.43%
CAATACAGTTAACTATT-GGAGG   d1   45.59%
CAATACAGTTAACTAT--GGAGG   d2   8.90%
CAATACAGTTAACT----GGAGG   d4   4.08%
```

Fig. S1 Somatic mutation types and frequencies in *N. benthamiana* and *N. tabacum* plant tissues infected with EMDV-tgtRNA-Cas9 vectors. The target site sequences of *PDS* and PAMs (labeled in red) are shown. Dashes denote nucleotide deletions, and letter in blue indicate inserted base. The numbers of nucleotide deletions (d#) or insertions (i#) are shown on the right side of each sequence, followed by the percentages of reads with this mutation type. Minor mutation types with reads below 4% are not shown. WT, wild-type sequence.

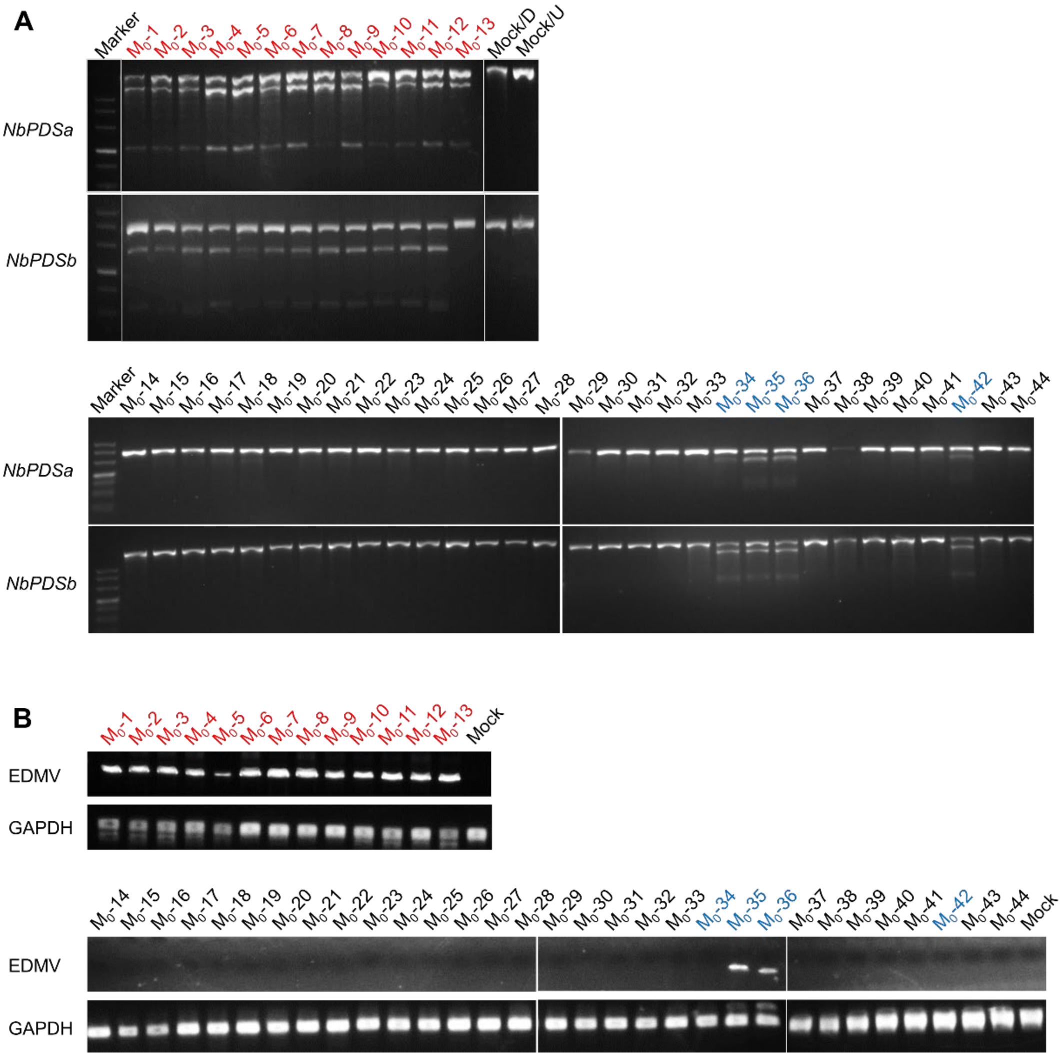

Fig. S2 Detection of target site mutations and the presence of EMDV vector in regenerated *N. benthamiana* plants. **A** Detection of targeted mutations in M_0 plantlets using T7E1 assay. DNA fragments encompassing the PDS2 target locus were amplified from individual regenerant by PCR and mixed in equal quantity with an DNA sample extracted from wild-type plants, followed by digestion with T7E1. DNA samples containing target site mutations would form mismatches upon annealing with the control DNA sample, thus being susceptible to T7E1 digestion. Albino line numbers are labeled in red, green line without and with mutations in black and blue, respectively. Mock/U and Mock/D denote mock-infected plants without or with restriction digestion, respectively. Marker, ladder DNA. **B** RT-PCR detection of EMDV vector using EMDV-specific primers listed in Table S4. Amplification of *GAPDH* mRNA serves as internal controls.

Table S1 Summary of the phenotypes and *PDS* genotypes of *N. benthamiana* and *N. tabacum* M_0 lines regenerated from virus-infected tissues

Line No.	Phenotype	Genotype			Virus
		Mutation type (*PDSa*/*b*)	Zygosity (*PDSa*/*b*)	Genotype	
N. benthamiana					
M_0-1	Albino	d1d2/d5d5	Bi/Ho	*aabb*	+
M_0-2		d1d1/d2d11	Ho/Bi	*aabb*	+
M_0-3		d1d1/d2d11	Ho/Bi	*aabb*	+
M_0-4		d4d5/d1i1	Bi/Bi	*aabb*	+
M_0-5		d4d5/d1i1	Bi/Bi	*aabb*	+
M_0-6		d1d1/d1i1	Ho/Bi	*aabb*	+
M_0-7		d4d5/d1d1	Bi/Ho	*aabb*	+
M_0-8		d4d198/d1d2	Bi/Bi	*aabb*	+
M_0-9		d1d4/d1d5	Bi/Bi	*aabb*	+
M_0-10		d1d1/d1i1	Ho/Bi	*aabb*	+
M_0-11		d1d1/d4d4	Ho/Ho	*aabb*	+
M_0-12		d1d8/d2d10	Bi/Bi	*aabb*	+
M_0-13		i1d10/d4d8	Bi/Bi	*aabb*	+
M_0-14	Normal	WT	WT	*AABB*	–
M_0-15		WT	WT	*AABB*	–
M_0-16		WT	WT	*AABB*	–
M_0-17		WT	WT	*AABB*	–
M_0-18		WT	WT	*AABB*	–
M_0-19		WT	WT	*AABB*	–
M_0-20		WT	WT	*AABB*	–
M_0-21		WT	WT	*AABB*	–
M_0-22		WT	WT	*AABB*	–
M_0-23		WT	WT	*AABB*	–
M_0-24		WT	WT	*AABB*	–
M_0-25		WT	WT	*AABB*	–
M_0-26		WT	WT	*AABB*	–
M_0-27		WT	WT	*AABB*	–
M_0-28		WT	WT	*AABB*	–
M_0-29		WT	WT	*AABB*	–
M_0-30		WT	WT	*AABB*	–
M_0-31		WT	WT	*AABB*	–
M_0-32		WT	WT	*AABB*	–
M_0-33		WT	WT	*AABB*	–
M_0-34		WTd1/WTd1	He/He	*AaBb*	–
M_0-35		d3d4/d1d1	Bi/Ho	*aabb*	+
M_0-36		d1WT/d1d1	He/Bi	*aAbb*	+
M_0-37		WT	WT	*AABB*	–
M_0-38		WT	WT	*AABB*	–

M_0-39		WT	WT	*AABB*	–
M_0-40		WT	WT	*AABB*	–
M_0-41		WT	WT	*AABB*	–
M_0-42		d4d4/d3d1	Ho/Bi	*aabb*	–
M_0-43		WT	WT	*AABB*	–
M_0-44		WT	WT	*AABB*	–
N. tabacum					
M_0-1	Albino	d1d4/d1d4	Bi/Bi	*aabb*	+
M_0-2		d1i1/d1i1	Bi/Bi	*aabb*	+
M_0-3		d1i2/d1d1	Bi/Ho	*aabb*	+
M_0-4		d1d5/d1d5	Bi/Bi	*aabb*	+
M_0-5		d1d1/d1d1	Ho/Ho	*aabb*	–
M_0-6	Normal	WT	WT	*AABB*	–
M_0-7		WT	WT	*AABB*	–
M_0-8		WT	WT	*AABB*	–
M_0-9		WT	WT	*AABB*	–
M_0-10		WT	WT	*AABB*	–
M_0-11		WT	WT	*AABB*	–
M_0-12		WT	WT	*AABB*	–
M_0-13		WT	WT	*AABB*	–
M_0-14		WT	WT	*AABB*	–
M_0-15		WT	WT	*AABB*	–
M_0-16		WTd1/WTd1	He/He	*AaBb*	–
M_0-17		WT	WT	*AABB*	–
M_0-18		WT	WT	*AABB*	–
M_0-19		WT	WT	*AABB*	–
M_0-20		d1d4/WTd1	Bi/He	*aaBb*	–
M_0-21		WT	WT	*AABB*	–
M_0-22		WT	WT	*AABB*	–
M_0-23		WTd1/WTd1	He/He	*AaBb*	+
M_0-24		WTd4/WTWT	He/WT	*AaBB*	–
M_0-25		WT	WT	*AABB*	–
M_0-26		WT	WT	*AABB*	–
M_0-27		WT	WT	*AABB*	–
M_0-28		WT	WT	*AABB*	–
M_0-29		WT	WT	*AABB*	–
M_0-30		WT	WT	*AABB*	–
M_0-31		WT	WT	*AABB*	–
M_0-32		WT	WT	*AABB*	–
M_0-33		WT	WT	*AABB*	–
M_0-34		WT	WT	*AABB*	–
M_0-35		WT	WT	*AABB*	–

Note: WT, wild-type sequence; d# and i#, # of base pairs deletion and insertion. Green letters indicate in-frame deletions. Ho, homozygote; Bi, bi-allelic, He, heterozygote (He).

Table S2 Summary of the *BBL* genotypes of *N. tabacum* M_0 lines regenerated from virus-infected tissues

Line No.	*BBLa*		*BBLb*		*BBLc*		*BBLd1*		*BBLd2*		*BBLe*		Genotype
	Mutation	Zygosity	Mutation	Zygosity	Mutation	Zygosity	Mutation	Zygosity	Mutation	Zygosity	Mutation	Zygosity	
M_0-#1	d2i1	Bi	i1i1	Ho	d1i1	Bi	d2i1	Bi	d1i1	Bi	d1ad1b	Bi	*aa/bb/cc/d1d1/d2d2/ee*
M_0-#2	WT	WT	WT	WT	WT	WT	WT	WT	WT	WT	WT	WT	*AA/BB/CC/D1D1/D2D2/EE*
M_0-#3	d2i1	Bi	d2i1	Bi	d1i1	Bi	d1ad1b	Bi	d2d3	Bi	d2i1	Bi	*aa/bb/cc/d1d1/d2d2/ee*
M_0-#5	WTd1	He	WTi1	He	WTi1	Chi	WTi1	He	WTd2	He	d4i1	Bi	*Aa/Bb/CC/D1d1/D2d2/ee*
M_0-#6	d3i1	Bi	d1d1	Ho	d1d14	Bi	d1d27	Bi	d2d2	Ho	d6i1	Bi	*aa/bb/cc/d1d1/d2d2/ee*
M_0-#7	WT	WT	WT	WT	WT	WT	WT	WT	WT	WT	WT	WT	*AA/BB/CC/D1D1/D2D2/EE*
M_0-#8	WT	WT	WT	WT	WT	WT	WT	WT	WT	WT	WT	WT	*AA/BB/CC/D1D1/D2D2/EE*
M_0-#9	WT	WT	WT	WT	WT	WT	WT	WT	WT	WT	WT	WT	*AA/BB/CC/D1D1/D2D2/EE*
M_0-#10	WT	WT	WT	WT	WT	WT	WT	WT	WT	WT	WT	WT	*AA/BB/CC/D1D1/D2D2/EE*
M_0-#11	WT	WT	WT	WT	WT	WT	WT	WT	WT	WT	WT	WT	*AA/BB/CC/D1D1/D2D2/EE*
M_0-#12	WT	WT	WT	WT	WT	WT	WT	WT	WT	WT	WT	WT	*AA/BB/CC/D1D1/D2D2/EE*
M_0-#13	WT	WT	WT	WT	WT	WT	WT	WT	WTi1	He	i1ai1b	Bi	*AA/BB/CC/D1D1/D2d2/ee*
M_0-#14	d2i1	Bi	d2i1	Bi	d9i1	Bi	d1ad1b	Bi	d2d3	Bi	d2i1	Bi	*aa/bb/cc/d1d1/d2d2/ee*
M_0-#15	WT	WT	WT	WT	WT	WT	WT	WT	WT	WT	WT	WT	*AA/BB/CC/D1D1/D2D2/EE*
M_0-#17	WT	WT	WT	WT	WT	WT	WT	WT	WT	WT	WT	WT	*AA/BB/CC/D1D1/D2D2/EE*
M_0-#18	WT	WT	WT	WT	WT	WT	WT	WT	WT	WT	WT	WT	*AA/BB/CC/D1D1/D2D2/EE*
M_0-#19	WT	WT	WT	WT	WT	WT	WT	WT	WT	WT	WT	WT	*AA/BB/CC/D1D1/D2D2/EE*
M_0-#20	WT	WT	WT	WT	WT	WT	WT	WT	WT	WT	WT	WT	*AA/BB/CC/D1D1/D2D2/EE*
M_0-#21	WT	WT	WT	WT	WT	WT	WT	WT	WT	WT	WT	WT	*AA/BB/CC/D1D1/D2D2/EE*
M_0-#23	d2i1	Bi	i1ai1b	Bi	d2i1	Bi	d2d8	Bi	d2i1	Bi	d16i1	Bi	*aa/bb/cc/d1d1/d2d2/ee*
M_0-#24	i1i1	Ho	d1i1	Bi	d1i1	Bi	d1i1	Bi	i1i1	Ho	i1i1	Ho	*aa/bb/cc/d1d1/d2d2/ee*
M_0-#25	WT	WT	WT	WT	WT	WT	WT	WT	WT	WT	WT	WT	*AA/BB/CC/D1D1/D2D2/EE*
M_0-#26	WT	WT	WT	WT	WT	WT	WT	WT	WT	WT	WT	WT	*AA/BB/CC/D1D1/D2D2/EE*
M_0-#27	WT	WT	WT	WT	WT	WT	WT	WT	WT	WT	WT	WT	*AA/BB/CC/D1D1/D2D2/EE*

Table S3 Summary of the *BBL* genotypes of *N. tabacum* M1 lines derived from M_0-#5 and M_0-#24

M1 line No.	*BBLa*		*BBLb*		*BBLc*		*BBLd1*		*BBLd2*		*BBLe*		Genotype
	Mutation	Zygosity	Mutation	Zygosity	Mutation	Zygosity	Mutation	Zygosity	Mutation	Zygosity	Mutation	Zygosity	
#5-006	d1d1	Ho	i1i1	Ho	WT	WT	i1i1	Ho	d2d2	Ho	d4d4	Ho	*aa/bb/CC/d1d1/d2d2/ee*
#5-090	d1d1	Ho	i1i1	Ho	WT	WT	i1i1	Ho	d2d2	Ho	d4d4	Ho	
#5-101	d1d1	Ho	i1i1	Ho	WT	WT	i1i1	Ho	d2d2	Ho	d4d4	Ho	
#5-041	d1d1	Ho	i1i1	Ho	WT	WT	i1i1	Ho	WT	WT	d4d4	Ho	*aa/bb/CC/d1d1/D2D2/ee*
#5-216	d1d1	Ho	i1i1	Ho	WT	WT	i1i1	Ho	WT	WT	d4d4	Ho	
#5-358	d1d1	Ho	i1i1	Ho	WT	WT	i1i1	Ho	WT	WT	d4d4	Ho	
#5-371	d1d1	Ho	i1i1	Ho	WT	WT	i1i1	Ho	WT	WT	d4d4	Ho	
#5-012	d1d1	Ho	i1i1	Ho	WT	WT	WT	WT	d2d2	Ho	d4d4	Ho	*aa/bb/CC/D1D1/d2d2/ee*
#5-186	d1d1	Ho	i1i1	Ho	WT	WT	WT	WT	d2d2	Ho	d4d4	Ho	
#5-016	d1d1	Ho	i1i1	Ho	WT	WT	WT	WT	WT	WT	d4d4	Ho	*aa/bb/CC/D1D1/D2D2/ee*
#5-374	d1d1	Ho	i1i1	Ho	WT	WT	WT	WT	WT	WT	d4d4	Ho	
#5-353	d1d1	Ho	WT	WT	WT	WT	i1i1	Ho	d2d2	Ho	i1i1	Ho	*aa/BB/CC/d1d1/d2d2/ee*
#5-418	d1d1	Ho	WT	WT	WT	WT	i1i1	Ho	d2d2	Ho	i1i1	Ho	
#5-434	d1d1	Ho	WT	WT	WT	WT	i1i1	Ho	d2d2	Ho	i1i1	Ho	
#5-440	d1d1	Ho	WT	WT	WT	WT	i1i1	Ho	d2d2	Ho	i1i1	Ho	
#5-280	d1d1	Ho	WT	WT	WT	WT	i1i1	Ho	WT	Ho	i1i1	Ho	*aa/BB/CC/d1d1/D2D2/ee*
#5-142	d1d1	Ho	WT	WT	WT	WT	WT	WT	d2d2	Ho	i1i1	Ho	*aa/BB/CC/D1D1/d2d2/ee*
#5-315	d1d1	Ho	WT	WT	WT	WT	WT	WT	d2d2	Ho	i1i1	Ho	
#5-195	d1d1	Ho	WT	WT	WT	WT	WT	WT	WT	WT	i1i1	Ho	*aa/BB/CC/D1D1/D2D2/ee*
#5-259	d1d1	Ho	WT	WT	WT	WT	WT	WT	WT	WT	i1i1	Ho	
#5-329	d1d1	Ho	WT	WT	WT	WT	WT	WT	WT	WT	i1i1	Ho	
#5-307	WT	WT	i1i1	Ho	WT	WT	i1i1	Ho	d2d2	Ho	d4d4	Ho	*AA/bb/CC/d1d1/d2d2/ee*
#5-148	WT	WT	i1i1	Ho	WT	WT	i1i1	Ho	WT	WT	d4d4	Ho	*AA/bb/CC/d1d1/D2D2/ee*

#5-227	WT	WT	i1i1	Ho	WT	WT	WT	WT	d2d2	Ho	d4d4	Ho	*AA/bb/CC/D1D1/d2d2/ee*
#5-105	WT	WT	i1i1	Ho	WT	WT	WT	WT	WT	WT	d4d4	Ho	*AA/bb/CC/D1D1/D2D2/ee*
#5-128	WT	WT	i1i1	Ho	WT	WT	WT	WT	WT	WT	d4d4	Ho	
#5-132	WT	WT	i1i1	Ho	WT	WT	WT	WT	WT	WT	d4d4	Ho	
#5-222	WT	WT	i1i1	Ho	WT	WT	WT	WT	WT	WT	d4d4	Ho	
#5-271	WT	WT	i1i1	Ho	WT	WT	WT	WT	WT	WT	d4d4	Ho	
#5-407	WT	WT	i1i1	Ho	WT	WT	WT	WT	WT	WT	d4d4	Ho	
#5-050	WT	WT	WT	WT	WT	WT	i1i1	Ho	d2d2	Ho	i1i1	Ho	*AA/BB/CC/d1d1/d2d2/ee*
#5-245	WT	WT	WT	WT	WT	WT	i1i1	Ho	d2d2	Ho	i1i1	Ho	
#5-250	WT	WT	WT	WT	WT	WT	i1i1	Ho	d2d2	Ho	i1i1	Ho	
#5-137	WT	WT	WT	WT	WT	WT	i1i1	Ho	WT	WT	i1i1	Ho	*AA/BB/CC/d1d1/D2D2/ee*
#5-073	WT	WT	WT	WT	WT	WT	WT	WT	WT	WT	i1i1	Ho	*AA/BB/CC/D1D1/D2D2/ee*
#5-334	WT	WT	WT	WT	WT	WT	WT	WT	WT	WT	i1i1	Ho	
#24-018	i1i1	Ho	d1d1	Ho	i1i1	Ho	d1d1	Ho	i1i1	Ho	i1i1	Ho	*aa/bb/cc/d1d1/d2d2/ee*
#24-019	i1i1	Ho	d1d1	Ho	d1d1	Ho	i1i1	Ho	i1i1	Ho	i1i1	Ho	
#24-026	i1i1	Ho	d1d1	Ho	i1i1	Ho	i1i1	Ho	i1i1	Ho	i1i1	Ho	

Table S4 List of primers used in plasmid construction and RT-PCR

Primer	Sequence (5' → 3')	Usage
pGD/Bsp119I/F	acaaatctatctctggatccTTCGAAAGAATGGCAGATAATTTCC	Construction of EMDV-tgtRNA-Cas9
NXJ/I/R	cactggtgctttgttGGTGTGTTGGGTTTTTATTAAAGG	
sgRNA/F	AACAAAGCACCAGTGGTCTAG	
sgRNA/AarI/R	aggtgttggtagtagCGACTCCATGCACCTGCTTGTTGCACCAGCCGGGAATC	
sgRNA/AarI/F	ctactaccaacacctGCGAACGTTTTAGAGCTAGAAATAGCAAGTTAAAATAAG	
sgRNA/R	TGCACCAGCCGGGAATC	
NXJ/II/F	ttcccggctggtgcaGATGATTTCTAATTTCAATTGCATGTG	
NXJ/II/R	gtccttatagtccatGGTGTGTTGGGTTTTTATTAAAGG	
Cas9/F	ATGGACTATAAGGACCACGACG	
Cas9/R	TTACTTTTTCTTTTTTGCCTGGCC	
NXJ/III/F	aaaaagaaaaagtaaGATGATTTCTAATTTCAATTGCATGTG	
pGD/XmaJI/R	tttgaacgagctctgtcgacCCTAGGTGGAGCTCTCACATAAG	
PDS1/F	tgcaTTGGTAGTAGCGACTCCATG	Golden Gate-based cloning of PDS1 gRNA
PDS1/R	aaacCATGGAGTCGCTACTACCAA	
PDS2/F	tgcaCAATACAGTTAACTATTTGG	Golden Gate-based cloning of PDS2 gRNA
PDS2/R	aaacCCAAATAGTTAACTGTATTG	
BBL1/F	tgcaGAAATCAGAGTAAGGTGCGG	Golden Gate-based cloning of BBL gRNA
BBL1/R	aaacCCGCACCTTACTCTGATTTC	
N/F	AATCTGCTCTTGATGATGTCAAGC	RT-PCR detection of EMDV
N/R	AGGTGGATGGGTTGTTCTTGATAG	
GAPDH/F	TGGAGAGGTGGAAGAGCTG	RT-PCR internal control
GAPDH/R	CCCTCTGATTCCTCCTTGATTG	

Note: Sequences shown in lowercase letters are designed to facilitate In-Fusion or Golden Gate cloning.

Table S5 List of oligos used for HTS, Sanger sequencing, and T7E1 assay

**Oligo**	**Sequence (5'→3')**	**Usage**
HTS/NbPDS1/F	ggagtgagtacggtgtgcGCTTATCTTTGGAGCTCGAGG	HTS of NbPDS1 locus
HTS/NbPDS1/R	gagttggatgctggatggTCAATGCAGACTACCTGAATGG	
HTS/NbPDS2/F	ggagtgagtacggtgtgcCTTGCCATTCAGGTAGTCTGC	HTS of NbPDS2 locus
HTS/NbPDS2/R	gagttggatgctggatggAATCACCTGCACCAGCAATAAC	
HTS/NtPDS2/F	ggagtgagtacggtgtgcGTTAAGGATTCGTACTCCCAGT	HTS of NtPDS1 locus
HTS/NtPDS2/R	gagttggatgctggatggCCTGCACCAGCAATAACAATC	
HTS/NtBBLa/b/c/F	ggagtgagtacggtgtgcGAGGAGCTCGTGAGCACCA	HTS of NtBBLa/b/c/d1/d2/e/ loci
HTS/NtBBLa/b/c/e/R	gagttggatgctggatggAATTTGGCCAATTGTTGCGC	
HTS/NtBBLd1/d2/F	ggagtgagtacggtgtgcAAGGAGCAGCTGGTGAG	
HTS/NtBBLd1/d2/R	gagttggatgctggatggAGTCTGGCCAAGTGTAGCGC	
HTS/Nt BBLe/F	ggagtgagtacggtgtgcAAAGAGGAGCTCGTGAG	
NtBBLa/F	TTTCATGCCGAAACCAACCT	Sanger sequencing of BBLa amplicon
NtBBLa/R	GAATTTTCCAGGCATAAACAATG	
NtBBLb/F	AGGTGGAGTTGCAAATCTTTA	Sanger sequencing of BBLb amplicon
NtBBLb/R	ATAATTCCCCAATTTCCACCT	
NtBBLc/F	CAGAATCTTCGATTTGCAGCATCTAA	Sanger sequencing of BBLc amplicon
NtBBLc/R	TGTTACGATTTTGGGCACTTTCAC	
NtBBLd1/F	AATCTCCGATTCGCAGCGTG	Sanger sequencing of BBLd1 amplicon
NtBBLd1/R	GTCCATATTTTCTGGATAAAAATCCGT	
NtBBLd2/F	AGTCAGTAACTTCTCTGTTTAT	Sanger sequencing of BBLd2 amplicon
NtBBLd2/R	AAATCCATGAACGTCACTGG	
NtBBLe/F	ACTTTCCTCTTTGCTAGTGTT	Sanger sequencing of BBLe amplicon
NtBBLe/R	ACCGTCCTTCAGCATCAATT	
NbPDSa/F	CTTGATTTTGTGGGTGAAGGC	NbPDSa T7E1 assay
NbPDSa/R	ACAGTTAATCAGAAAAGAATGTACAGG	
NbPDSb/F	AGTGTGATGCTGAATTTATGATCAC	NbPDSa T7E1 assay
NbPDSb/R	AAATCACTCCTAATCTAATCAGTTGG	

Note: Sequences shown in lowercase letters are the adaptor sequences ‘ggagtgagtacggtgtgc’ (forward primers) and ‘gagttggatgctggatgg’ (reverse primers) that serve as annealing sites for the index primers in the second round PCR.
